# Sex differences in innate anti-viral immune responses to respiratory viruses and in their clinical outcomes in a birth cohort study

**DOI:** 10.1038/s41598-021-03044-x

**Published:** 2021-12-09

**Authors:** Eteri Regis, Sara Fontanella, Lijing Lin, Rebecca Howard, Sadia Haider, John A. Curtin, Michael R. Edwards, Magnus Rattray, Angela Simpson, Adnan Custovic, Sebastian L. Johnston

**Affiliations:** 1 Respiratory Medicine and Allergy, National Heart and Lung Institute, Imperial College London, Norfolk Place, London, W2 1PG UK; 2 Faculty of Biology, Medicine and Health, University of Manchester, Manchester, M13 9PT UK; 3 Division of Infection, Immunity and Respiratory Medicine, Faculty of Biology, Medicine and Health, Manchester Academic Health Sciences Centre, University of Manchester and University Hospital of South Manchester NHS Foundation Trust, Manchester, UK

**Keywords:** Immunology, Infectious diseases, Innate immunity

## Abstract

The mechanisms explaining excess morbidity and mortality in respiratory infections among males are poorly understood. Innate immune responses are critical in protection against respiratory virus infections. We hypothesised that innate immune responses to respiratory viruses may be deficient in males. We stimulated peripheral blood mononuclear cells from 345 participants at age 16 years in a population-based birth cohort with three live respiratory viruses (rhinoviruses A16 and A1, and respiratory syncytial virus) and two viral mimics (R848 and CpG-A, to mimic responses to SARS-CoV-2) and investigated sex differences in interferon (IFN) responses. IFN-α responses to all viruses and stimuli were 1.34–2.06-fold lower in males than females (*P* = 0.018 −  < 0.001). IFN-β, IFN-γ and IFN-induced chemokines were also deficient in males across all stimuli/viruses. Healthcare records revealed 12.1% of males and 6.6% of females were hospitalized with respiratory infections in infancy (*P* = 0.017). In conclusion, impaired innate anti-viral immunity in males likely results in high male morbidity and mortality from respiratory virus infections.

## Introduction

Respiratory viral infections are among most common causes of severe illness and death globally^1^, and males and females differ substantially in both prevalence and severity of viral infections^2^. For example, the overall infection rates for respiratory syncytial virus (RSV), which is one of the most common causes of acute respiratory infection worldwide^3^, are higher among boys, and male sex is a risk factor for severe RSV bronchiolitis requiring hospitalization^4^ (with ~ 40% increase in risk^5^). Sex differences in the incidence, severity, hospitalization rates and mortality from both pandemic and seasonal Influenza are also well documented, and males have consistently been reported to be at higher risk^6–8^. Male predominance was reported in rates of hospitalization due to lower respiratory tract infections (LRTIs) of all causes in danish populations under the age of 25 years^9^. These profound sex differences have been highlighted by the immense global impact of COVID-19^10^, in which risk factors for mortality include older age, the presence of comorbid conditions, and male sex^11,12^. For example, a large UK study reported that COVID-19-related death was associated with being male with a hazard ratio of 1.59^13^, and similar sex differences were reported from other parts of the world^14,15^. Intensive Care Unit (ICU) admission^16^, hospital admissions^17^ and case identification in population screening^18^ also report a male preponderance.

Sex differences have also been described in immunity to multiple vaccines in both children and adults^19^. A randomized study of humoral immune responses to trivalent inactivated influenza vaccine reported that healthy women aged 18–64 years generated a more robust protective antibody response, and women responded to a half dose of the vaccine with an antibody response equivalent to the full dose in men^20^.

The association of infection rates and poor outcomes with male sex is consistent across different respiratory viruses, but the mechanisms explaining excess morbidity in males are poorly understood^21^.

One study has shown that pre-menopausal females have stronger adaptive immune response to rhinovirus (RV) A16 infection in peripheral blood mononuclear cells (PBMCs) than men and older females, however they saw no sex differences in innate immune responses to RV-A16^22^.

We propose that the consistency of sex-related differences across most respiratory viruses may reflect inherent differences in innate immune responses to viruses between the sexes. Innate immune responses, mediated by anti-viral interferon (IFN) production by virus-infected cells will be critical in protection against viruses, especially those that humans have never previously encountered. However, little is known about the variation within the human immune system in patterns of innate immune response to viruses at a population level. We hypothesise that the adverse outcomes for males reported in relation to respiratory virus infections may be related to deficient innate immune responses to viruses in males relative to females. To address our hypothesis, within the setting of a population-based birth cohort study, we investigated IFN and IFN-induced chemokine responses of PBMCs from 16-year-old male and female participants after stimulation with three common respiratory viruses and two viral mimics. We ascertained the frequency of hospital admissions with LRTIs and bronchiolitis in early life among males and females in our cohort, to confirm that previously observed sex differences hold true in this study population. Finally, to determine whether deficient IFN responses related to severe illness frequencies, we investigated frequencies of hospital admissions with LRTIs and bronchiolitis in participants with the lowest IFN responses (below the 15th percentile of the entire population) and in those above this percentile.

## Results

### Frequencies of early life hospitalizations for severe respiratory viral illnesses in cohort participants

We first investigated whether frequencies of early-life LRTIs (which are almost exclusively viral in aetiology) differed between sexes in our cohort to ensure the study population was representative of the excess morbidity with virus infections observed in males in the general population. We focussed on early life, as this is the life stage when innate immune responses will be most important, as the very young will have had little opportunity to develop memory responses. A total of 916 study participants had transcribed data from primary care records; results are presented in Table 1. There was a significant difference between the sexes in the proportion of children admitted to hospital with LRTI in the first year of life, with 56/498 (11.2%) of males and 28/418 (6.7%) of females being hospitalized (*P* = 0.018). In the second year of life, 30/498 (6%) of males but only 5/4181(1.2%) of females were admitted to hospital with LRTI (*P* < 0.001). We observed a similar pattern for hospitalizations for physician-confirmed bronchiolitis (males 43/498 [8.6%] vs. females 16/418 [3.8%], *P* = 0.003) and RSV-positive bronchiolitis (males 20/498 [4%] vs. females 7/418 [1.7%], *P* = 0.037). These sex differences in the risk of LRTI hospital admissions remained stable from infancy to mid-school age, with males being twice as likely to be admitted as females (odds ratio [OR] 2.01, 95% CI 1.39–2.91, *P* < 0.001). We also compared frequencies of early-life LRTIs between sexes in participants who provided blood samples for the assessment of innate anti-viral immune responses at age 16 years (Table S1). We observed the same trends which we identified in the general population, although some of the associations failed to reach statistical significance due to a lower sample size. The difference in bronchiolitis was statistically significant, with 16 (8.2%) males and only 3 (2.6%) females being hospitalized (*P* = 0.045).

**Table 1:** Comparisons of lower respiratory tract infection (LRTI) hospital admissions, bronchiolitis and RSV positive bronchiolitis cases in female and male cohort participants.

	Total	%	Male	%	Female	%	*P*-value	OR	95% CI	*P*-value
LRTI admission in the first year of life	84/916	9.17	56/498	11.24	28/418	6.70	0.018	1.76	1.10–2.83	0.019
LRTI admission in the second year of life	35/916	3.82	30/498	6.02	5/418	1.20	< 0.001	5.29	2.04–13.77	0.001
LRTI admission in the first two years of life	108/916	11.79	77/498	15.46	31/418	7.42	< 0.001	2.28	1.47–3.54	< 0.001
LRTI admission by third year of life	125/916	13.65	89/498	17.87	36/418	8.61	< 0.001	2.31	1.53–3.48	< 0.001
Ever hospitalized with LRTI, age 0–8	151/916	16.48	103/498	20.68	48/418	11.48	< 0.001	2.01	1.39–2.91	< 0.001
RSV positive bronchiolitis	27/916	2.95	20/498	4.02	7/418	1.67	0.037	2.46	1.03–5.87	0.043
Bronchiolitis	59/916	6.44	43/498	8.63	16/418	3.83	0.003	2.37	1.32–4.28	0.004

Differences were assessed through χ^2^ and *Fishers exact test* for sample size > 5 and ≤ 5, respectively, and logistic regression analysis (reference group: males).

*OR* odds ratio, *95% CI* 95% confidence intervals.

### Innate anti-viral immune responses

**Figure 1:**
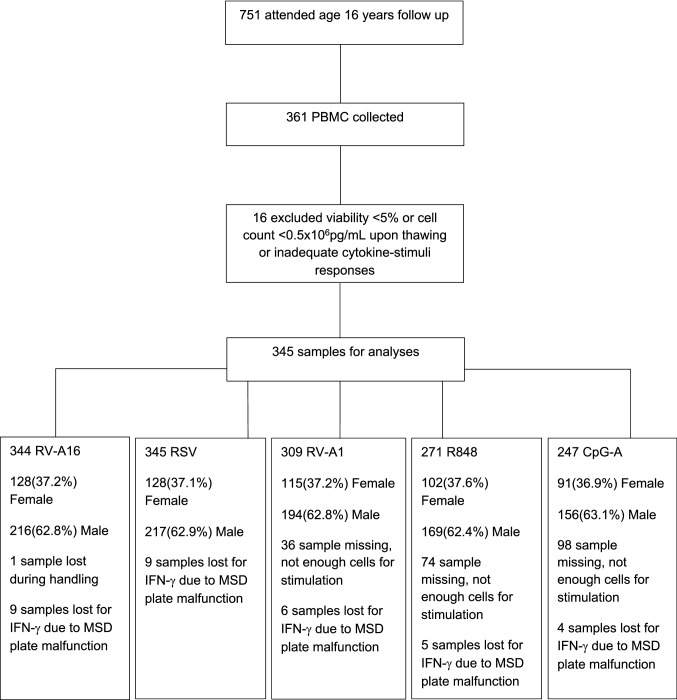
Participant flow and PBMC stimulation numbers for each stimulus at age 16 years.

Participant flow for the study of innate anti-viral immune responses is presented in Fig. 1. Of 751 participants who attended clinical follow-up at age 16 years, 361 provided blood samples. After quality control of PBMC cytokine response data (using methods exactly as reported in our previous studies on this cohort using PBMCs collected at age 11 years^23,24^, details in “Methods” section—*Data pre-processing*), we excluded data for 16 individuals. There were no differences in demographics, environmental exposures and clinical features between participants included in this analysis (n = 345) and those who were not (n = 406), either in the whole population or stratified by sex (Table S2).

The demographic and clinical characteristics of the 345 subjects included in the analysis are shown in Table 2. There were no significant differences between sexes in birth weight, relevant environmental exposures (including tobacco smoke exposure, position in sibship and pet ownership) or common respiratory diseases, such as wheezing and asthma. No significant differences were observed in cell viability between sexes (Figure S1).

**Table 2:** Demographic and clinical characteristics of the participants included in the study of innate anti-viral immune responses.

	Participants with cytokine data (n = 345)		
	Female	Male	*p*-value
	n (%)	n (%)	χ^2^
Ethnicity (Caucasian)	121/126 (96.0)	204/212 (96.2)	0.504
Younger siblings	112/217 (51.6)	70/128 (54.7)	0.581
Older siblings	62/128 (48.4)	121/215 (56.3)	0.159
Day care attendance	91/121 (75.2)	153/209 (73.2)	0.690
Maternal smoking (pregnancy)	9/128 (7.0)	19/212 (9.0)	0.530
Maternal smoking (current)	10/126 (7.9)	29/217 (13.4)	0.127
Maternal asthma	23/128 (18.0)	39/217 (18.0)	0.999
Paternal asthma	22/128 (17.2)	29/217 (13.4)	0.334
Dog ownership	40/127 (31.5)	80/216 (37.0)	0.298
Cat ownership	29/127 (22.8)	54/215 (25.1)	0.634
Current asthma	20/126 (18.3)	39/213 (18.3)	0.567
Current wheeze	18/126 (14.3)	33/215 (15.3)	0.790

	Mean (SD)	Mean (SD)	t-test
Age at follow up	16.0 (0.65)	16.1 (0.55)	0.763
Birth weight (kg)	3.42 (0.47)	3.51 (0.91)	0.242

Some data was lost due to individuals not attending a follow up visit or skipping the question.

### Induction of IFNs and IFN-induced chemokines

**Figure 2:**
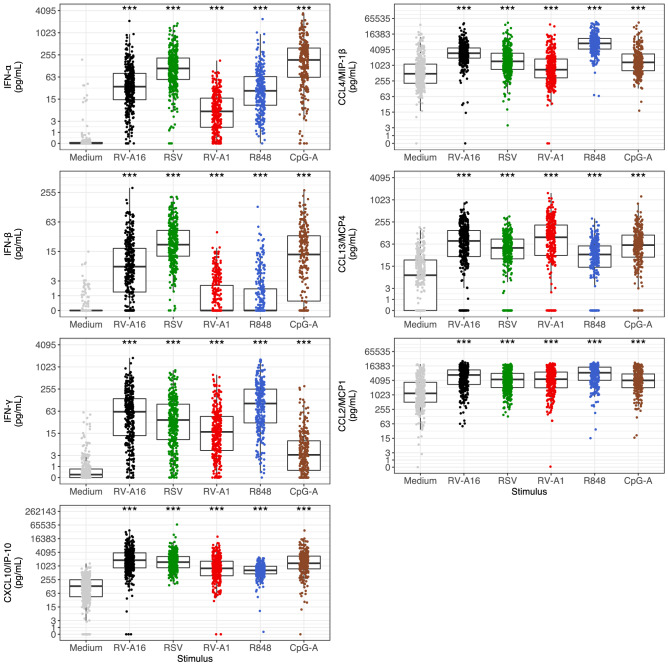
Patterns of PBMC IFN- and IFN-induced chemokine-induction by viral stimuli, compared to medium controls. Data were analysed using the Wilcoxon test. Benjamini–Hochberg correction was applied to account for multiple testing. Each dot represents an individual participant. Box plots represent the 25th and 75th percentiles, the line the median, with whiskers at the 10th and 90th percentiles. Data are presented in pg/mL. The *y* axis is plotted on a logarithmic scale. Significance levels: ***P < 0.001 compared to medium.

Induction of IFNs and IFN-induced chemokines in PBMCs in response to viral stimuli is shown in Fig. 2. All IFNs and IFN-induced chemokines were significantly induced (all *P* < 0.001) in response to all viral stimuli compared to medium control. The most potent inducers of IFN-α were CpG-A, RSV and RV-A16, with median concentrations of 184.7 pg/mL, 108.3 pg/mL and 34.0 pg/mL respectively compared to 0.0 pg/mL in medium control. RV-A1 and R848 also induced IFN-α, but to lesser degrees. Induction of IFN-β followed a very similar pattern to that of IFN-α, but with lower concentrations.

The most potent inducers of IFN-γ were R848, RV-A16 and RSV, with median concentrations of 102.1 pg/mL, 60.7 pg/mL and 35.7 pg/mL, respectively, compared to 0.2 pg/mL in medium control. RV-A1 and CpG-A also induced IFN-γ, but to lesser degrees.

The most potent inducers of the IFN-induced chemokine CXCL10/IP-10, were RV-A16, RSV and CpG-A, with median concentrations of 1841.0 pg/mL, 1515.2 pg/mL and 1343.4 pg/mL respectively, compared to 130.6 pg/mL in medium control samples. RV-A1 and R848 also induced CXCL-10/IP-10, but to lesser degrees.

The IFN-induced chemokines CCL2/MCP1, CCL4/MIP-1β and CCL13/MCP4 were also all induced by all viral stimuli, CCL2/MCP1 and CCL4/MIP-1β most potently by R848 and CCL13/MCP4 most potently by RV-A1.

### Differences between males and females in IFN and IFN-induced chemokine induction

Results of the comparisons between sexes are presented in Table 3.

**Table 3:** Differences between males and females in IFN and IFN-induced chemokine responses to viral stimuli.

Stimulus	Cytokine	Median log2 fold Induction [IQR]	Median log2 fold Induction [IQR]	*P* value	Adjusted *P* value	Female to Male ratio in fold induction
		Female, n = 128 (37.2%)	Male, n = 216 (62.8%)			
RV-A16	IFN-α	*10.24 [8.98–11.55]*	9.30 [7.88–10.53]	** < 0.001**	**0.001**	1.92
	IFN-β	*6.01 [4.66–7.26]*	4.98 [2.02–6.66]	** < 0.001**	**0.003**	2.04
	IFN-γ	*7.69 [6.04–8.90]*	6.87 [5.03–8.15]	**0.003**	**0.017**	1.77
	CCL4/MIP-1β	*2.13 [1.47–3.25]*	1.98 [0.96–2.98]	0.125	0.231	1.11
	CXCL10/IP-10	*4.15 [3.13–5.27]*	3.66 [2.36–4.95]	**0.010**	**0.041**	1.04
	CCL13/MCP4	*2.60 [1.19–3.85]*	2.10 [0.93–3.41]	0.076	0.149	1.41
	CCL2/MCP1	*2.09 [1.09–2.95]*	1.91 [0.95–2.92]	0.659	0.721	1.13

		Female n = 128 (37.1%)	Male n = 217 (62.9%)			
RSV	IFN-α	*11.66 [10.53–12.53]*	11.24 [9.91–12.21]	**0.018**	0.055	1.34
	IFN-β	*6.89 [5.83–7.49]*	6.62 [5.58–7.71]	0.583	0.658	1.21
	IFN-γ	*7.08 [5.88–8.14]*	6.54 [4.84–8.11]	0.062	0.142	1.45
	CCL4/MIP-1β	*1.60 [1.08–2.36]*	1.58 [0.98–2.20]	0.380	0.475	1.01
	CXCL10/IP-10	*3.67 [2.59–4.83]*	3.36 [2.38–4.62]	0.210	0.304	1.24
	CCL13/MCP4	*1.46 [0.42–2.51]*	1.12 [-0.11–2.13]	**0.019**	0.055	1.27
	CCL2/MCP1	*1.75 [0.93–2.53]*	1.64 [0.64–2.65]	0.799	0.848	1.08

		Female n = 115 (37.2%)	Male n = 194 (62.8%)			
RV-A1	IFN-α	*7.54 [5.68–8.92]*	6.91 [4.92–8.34]	**0.011**	**0.041**	1.55
	IFN-β	1.21 [1.21–3.61]	1.21 [1.21–3.16]	0.483	0.583	1.00
	IFN-γ	*6.08 [4.32–7.27]*	5.51 [3.69–6.83]	0.066	0.142	1.48
	CCL4/MIP-1β	*0.93 [0.43–1.41]*	0.71 [0.20–1.13]	**0.010**	**0.041**	1.16
	CXCL10/IP-10	*3.05 [1.94–4.17]*	2.75 [1.36–3.85]	**0.041**	0.104	1.23
	CCL13/MCP4	*3.39 [2.07–4.47]*	2.70 [0.67–3.80]	**0.002**	**0.017**	1.61
	CCL2/MCP1	*2.00 [1.09–2.77]*	1.84 [0.85–2.58]	0.217	0.304	1.12

		Female n = 102 (37.6%)	Male n = 169 (62.4%)			
R848	IFN-α	*9.91 [8.41–11.52]*	8.87 [7.47–10.26]	** < 0.001**	**0.002**	2.06
	IFN-β	1.53 [1.53–3.27]	1.53 [1.53–2.84]	0.158	0.261	1.00
	IFN-γ	*7.51 [5.84–9.14]*	7.11 [5.45–8.60]	0.164	0.261	1.32
	CCL4/MIP-1β	*3.58 [2.94–4.45]*	3.46 [2.56–4.42]	0.375	0.475	1.09
	CXCL10/IP-10	*2.15 [1.25–3.15]*	1.79 [1.04–2.82]	0.069	0.142	1.28
	CCL13/MCP4	*0.83 [-0.31–1.85]*	0.76 [-0.85–1.69]	0.239	0.322	1.05
	CCL2/MCP1	*2.26 [0.79–3.14]*	2.02 [0.97–3.04]	0.914	0.914	1.18

		Female n = 91 (36.9%)	Male n = 156 (63.1%)			
CpG-A	IFN-α	*12.6 [10.99–13.73]*	11.83 [10.47–13.38]	**0.015**	0.051	1.71
	IFN-β	*6.31 [2.86–8.10]*	5.71 [1.40–7.56]	0.155	0.261	1.52
	IFN-γ	*4.04 [2.29–5.17]*	3.03 [1.54–4.26]	**0.003**	**0.017**	2.01
	CCL4/MIP-1β	1.35 [0.91–1.91]	*1.43 [0.87–1.81]*	0.857	0.882	0.95
	CXCL10/IP-10	*4.02 [2.94–4.93]*	3.66 [2.71–4.67]	0.204	0.304	1.28
	CCL13/MCP4	*2.56 [1.70–3.53]*	2.12 [0.88–3.32]	**0.040**	0.104	1.36
	CCL2/MCP1	*1.66 [1.07–2.19]*	1.54 [0.89–2.07]	0.528	0.616	1.09

Data were analysed using the Wilcoxon test. *P* values and adjusted *P* values less than 0.05 are in bold. The group with the higher IFN and IFN-induced chemokine induction is highlighted in italic.

The induction of IFN-α was significantly higher in females than in males for all five viral stimuli (with *P*-values ranging from < 0.001 to 0.018). After adjustment for multiple testing, these differences remained statistically significant for RV-A16, RV-A1 and R848 (*P* = 0.001, 0.041 and 0.002 respectively), and marginal for RSV and CpG-A (*P* = 0.055 and 0.051). The magnitude of the differences observed for IFN-α ranged from a 1.34-fold greater induction of IFN-α in females than in males for RSV, to a 2.06-fold greater induction in females than in males for R848.

**Figure 3:**
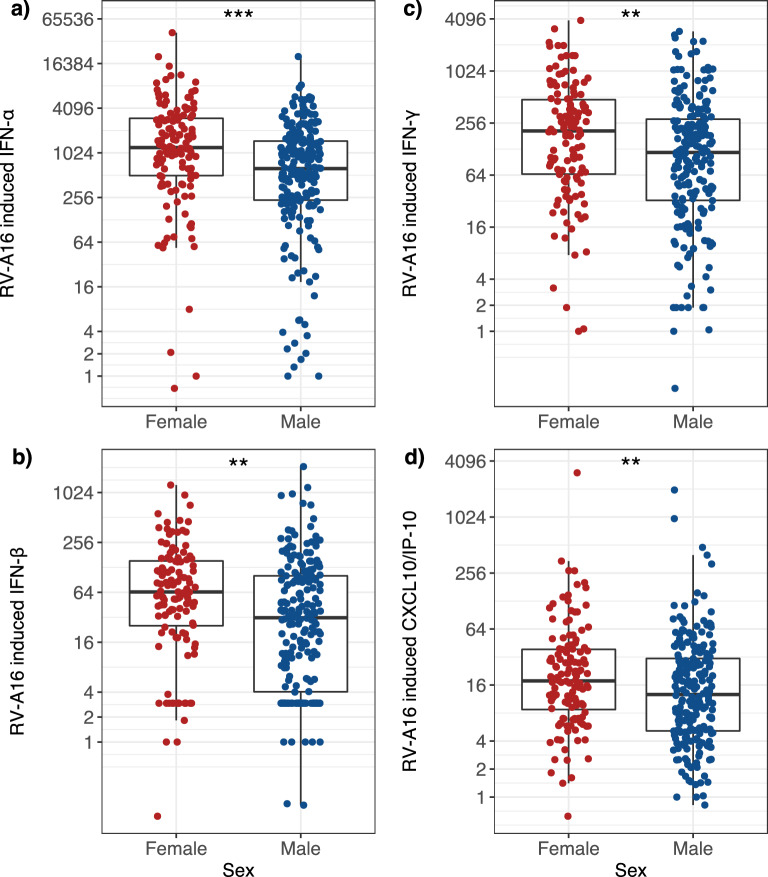
Females have significantly greater induction than males of type I and II IFNs and CXCL10/IP-10 in response to RV-A16. Box plots represent the 25th and 75th percentiles, the line the median, with whiskers at the 10th and 90th percentiles. Each dot represents an individual participant. Data were analysed using the Wilcoxon test. Adjusted *P* values: (**a**) *P* = 0.001, (**b**) *P* 0.003, (**c**) *P* = 0.017 and (**d**) *P* = 0.041. ‘***’ 0.001 ‘**’ 0.05 ‘*’ 0.1. Data are presented as fold induction. The y axis is plotted on a logarithmic scale.

The individual responses of each IFN and of CXCL10/IP-10 to RV-A16 stimulation are depicted in Fig. 3. For IFN-α, the induction in females was 1.92-fold greater than that in males (*P* 0.001 Table 3; individual data depicted in Fig. 3a). Similarly, females had 2.04-fold greater induction of IFN-β (*P* = 0.003, Table 3; Fig. 3b), 1.77-fold greater induction of IFN-γ (*P* = 0.017, Table 3; Fig. 3c) and 1.40-fold greater induction of CXCL10/IP-10 *(P* = 0.0, Table 3; Fig. 3d).

**Figure 4:**
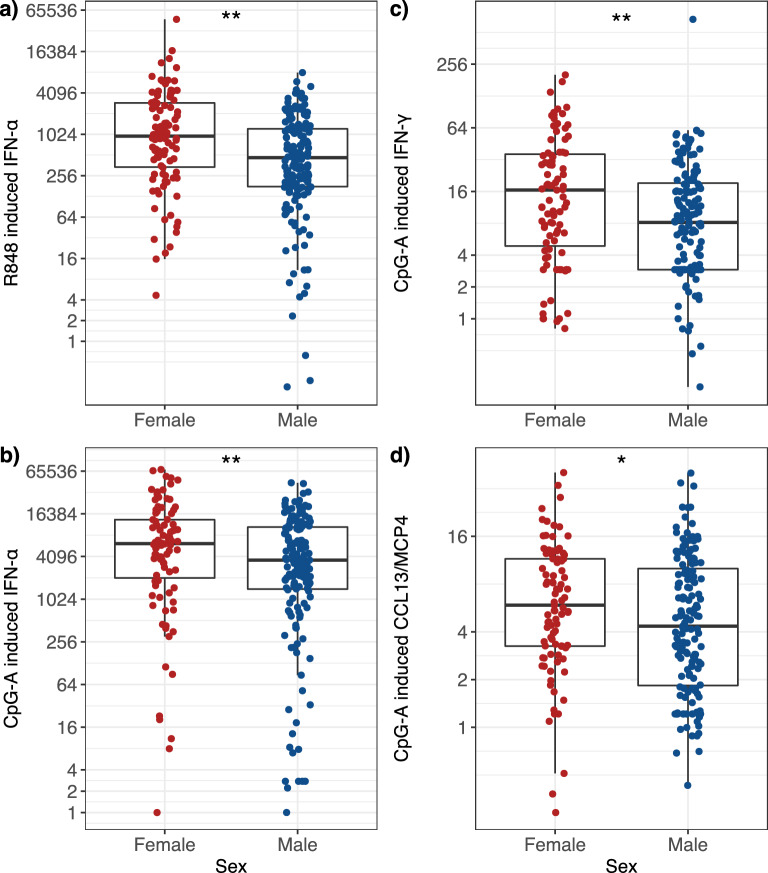
Females have significantly greater induction than males of type I and II IFNs and CCL13/MCP4 in response to the viral mimics R848 and CpG-A. Box plots represent the 25th and 75th percentiles, the line the median, with whiskers at the 10th and 90th percentiles. Each dot represents an individual participant. Data were analysed using the Wilcoxon test. Adjusted *P* values: (**a**) *P* = 0.002, (**b**) *P* = 0.051, (**c**) *P* = 0.017 and (**d**) *P* = 0.104. ‘***’ 0.001 ‘**’ 0.05 ‘*’ 0.1. Data are presented as fold induction. The y axis is plotted on a logarithmic scale.

Individual cytokine responses to viral mimics R848 and CpG-A (to mimic SARS-CoV-2) are shown in Fig. 4. Females had significantly greater induction of IFN-α in response to R848 stimulation (2.06-fold, *P* = 0.002, Table 3), and in response to CpG-A difference was marginal (1.71-fold, *P* = 0.051, Table 3; Fig. 4a,b). Females also had significantly greater induction of IFN-γ (twofold, *P* = 0.017, Table 3) in response to CpG-A (Fig. 4c,d).

Stimulation with RV-A1 also resulted in significantly greater induction in females than in males for IFN-α (1.55-fold, *P* = 0.041, Table 3; Figure S2a), CXCL10/IP-10 (1.23-fold, *P* = 0.041prior adjustment, Table 3; Figure S2b) though this difference was marginal after adjustment (*P* = 0.10, Table 3), CCL4/MIP-1β (1.16-fold, *P* = 0.041, Table 3; Figure S2c) and CCL13/MCP4 (1.61-fold, *P* = 0.017, Table 3; Figure S2d). Finally, in response to RSV stimulation, females had greater induction of IFN-α and CCL13/MCP4 (both *P* = 0.055, Table 3; Figure S2e,f).

The trend across all viral stimuli and responses was consistent; although many of the associations failed to reach statistical significance after correcting for multiple testing, almost all IFNs and IFN-induced chemokines had higher levels of induction in females compared to males: out of 35 stimulus-cytokine pairs, females exhibited higher induction than males in 32 pairs, with only one pair higher in males, and the two other pairs equal between sexes (Table 3).

Similar analyses were also performed on raw cytokine concentrations expressed in pg/mL. These data are shown in Table S3. The results were broadly similar to those analysed as fold-induction, with females again exhibiting significantly higher induction than males in 6/35 cytokine/stimulus pairs and trend toward higher induction in 26/35. There were no significant differences between sexes in unstimulated samples cultured with media alone.

### Patterns of IFN induction were consistent across stimuli

Alluvial plots of the individual IFN-α responses across the five stimuli are shown in Figure S3. More males were in the lower quartile, and more females were in the higher quartiles of the whole-population response. Within-individual patterns suggested that individuals tended to have consistent (either higher or lower) responses across the stimuli significantly more often than would be expected by chance, indicating that the strength of response across different viral stimuli is relatively stable within individuals.

### More males than females were in the lower percentiles of IFN induction

**Figure 5:**
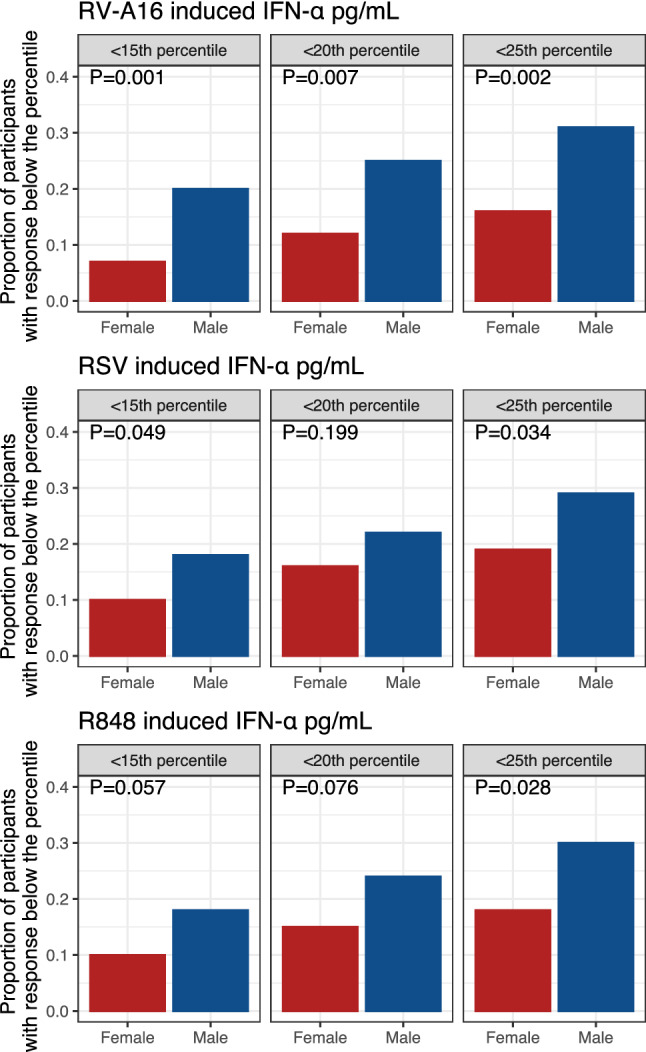
Proportions of males and females with IFN responses to RV-A16, RSV and R848 below the 15th, 20th and 25th percentiles of the entire population. *P*-values are derived using chi-squared test.

Since adverse clinical outcomes in viral infections in asthma are reported in those individuals with the weakest innate anti-viral responses^25^, we examined proportions of males and females whose innate anti-viral responses were below certain lower thresholds. We restricted this analysis to IFN-α, and low thresholds were defined as the 15th, 20th and 25th percentile of the response determined from the entire population. Results are shown in Figs. 4 and S5. Proportions of males having IFN-α responses to RV-A16, RSV and R848 below each threshold were significantly higher compared to females (Fig. 5). Similar trends were observed for response of IFN-α to RV-A1, and CpG-A (Figure S4), but these did not reach statistical significance.

### Frequencies of severe LRTIs were greater in participants below the 15th percentile of IFN induction

To determine whether low IFN responses were associated with adverse clinical outcomes in this cohort, frequencies of hospitalizations for severe viral respiratory illnesses in early-life were analyzed in relation to IFN-α responses to RV-A16 and RSV that were below or above the 15th percentile of the entire population. The frequency of LRTI and bronchiolitis hospital admissions was consistently 2–3 times higher in the participants < 15th percentile threshold compared to those > 15th percentile (Table 4), confirming that deficient IFN induction was indeed associated with increased frequencies of severe LRTIs in this cohort. The strength of the associations between low IFN production and the frequency of LRTI and bronchiolitis admissions were stronger in males than in females (Table 5).

**Table 4:** Comparisons of LRTI and bronchiolitis hospital admissions in participants with IFN-α responses to RV-A16 and RSV below and above the 15th percentile of the entire cohort.

Stimuli	Cytokine		< 15th percentile	%	> 15th percentile	%	*P*-value
RV-A16	IFN-⍺	LRTI admission in the first year of life	*7/47*	*14.9*	19/263	7.2	0.090
		Ever hospitalised for LRTI age 0–8	*13/47*	*27.7*	37/263	14.1	**0.020**
		Ever bronchiolitis	*7/47*	*14.9*	12/263	4.6	**0.007**
RSV	IFN-⍺	LRTI admission in the first year of life	*8/48*	*16.7*	18/263	6.8	**0.024**
		Ever hospitalised for LRTI age 0–8	*12/48*	*25.0*	38/263	14.5	0.067
		Ever bronchiolitis	*6/48*	*12.5*	13/263	4.94	**0.044**

Differences were assessed using χ^2^ tests. *P* values less than 0.05 are in bold. The group with the higher frequency of LRTI hospital admissions and bronchiolitis cases in early life is highlighted in italic.

**Table 5:** Comparisons of LRTI and bronchiolitis hospital admissions in participants with IFN-α responses to RV-A16 and RSV below and above the 15th percentile stratified by sex.

Stimuli	Cytokine		Woolf test for homogeneity	Male					Female				
			*P*-value	< 15th percentile	%	> 15th percentile	%	*P*-value	< 15th percentile	%	> 15th percentile	%	*P*-value
RV-A16	IFN-⍺	LRTI admission in the first year of life	0.968	*6/39*	*15.4*	12/155	7.7	0.211	*1/8*	*12.5*	7/108	6.5	0.446
		Ever hospitalised for LRTI age 0–8	0.895	*11/39*	*28.2*	24/155	15.5	0.100	*2/8*	*25.0*	13/108	12.0	0.276
		Ever bronchiolitis	0.671	*7/39*	*17.9*	9/155	5.8	**0.022**	0/8	0.0	*3/108*	*2.8*	1.000
RSV	IFN-⍺	LRTI admission in the first year of life	0.891	*6/35*	*17.1*	12/160	7.5	0.101	*2/13*	*15.4*	6/103	5.8	0.220
		Ever hospitalised for LRTI age 0–8	0.562	*10/35*	*28.6*	25/160	15.6	0.088	*2/13*	*15.4*	13/103	12.6	0.675
		Ever bronchiolitis	0.505	*6/35*	*17.1*	10/160	6.2	**0.045**	0/13	0.0	*3/103*	*2.9*	1.000

Differences stratified by sex were assessed using Fisher exact tests. *P* values less than 0.05 are in bold. The group with the higher frequency of LRTI hospital admissions and bronchiolitis cases in early life is highlighted in italic.

## Discussion

We hypothesized that the adverse outcomes in respiratory virus infections reported for males, which include higher mortality, ICU admissions, hospital admissions and case identification, may be related to deficient innate immune responses in males relative to females. Our analysis of the induction of three IFNs and four IFN-induced chemokines by five respiratory viruses/viral stimuli in PBMCs sampled from 16-year-old participants in a population-based birth cohort demonstrated that females produced approximately twice the concentrations of IFN-α and IFN-β in response to RV-A16 and of IFN-α in response to R848, 55% more IFN-α in response to RV-A1 and 71% and 52% more IFN-α and IFN-β in response to CpG-A. Responses in females were greater in 32 of 35 cytokine-stimulus pairs, including statistically significant differences in IFN-α induction in response to each viral stimulus, IFN-β induction in response to RV-A16, and IFN-γ induction in response to RV-A16 and CpG-A. These deficiencies in IFN responses in males were mirrored by significantly higher incidence of hospital admissions due to severe LRTIs in early life (which are almost exclusively viral in aetiology) among male participants in our cohort, as well as substantially higher prevalence of RSV-positive bronchiolitis, a disease with a peak incidence at 4.5 months of age^26^. Early life is the time during which innate immune responses will be most important, as young children will have had limited exposure to viruses and will therefore have little memory/acquired immunity.

To confirm that participants with the lowest IFN induction had adverse clinical outcomes in relation to the most common respiratory viral infections (RVs and RSV), we compared frequencies of hospitalizations for LRTIs and bronchiolitis (the great majority of which are caused by RVs and RSV) in participants with IFN-α induction in response to RV-A16 and RSV below the 15th percentile, with frequencies in participants with responses above the 15th percentile. These analyses confirmed that these adverse clinical outcomes were 2–3 times more frequent in participants with the lowest IFN induction. This lends credence to the biological and clinical relevance of our findings in relation to diseases where innate immune responses will be vitally important in protection against adverse outcomes, such as early life virus infections and novel zoonotic virus infections at any age, such as COVID-19.

Our study has strengths and limitations. Strengths include: (1) the study design (population-based birth cohort), which increases confidence that participants are representative of the general population; (2) the fact that respiratory illness ascertainment was carried out by personal transcription of healthcare records, thereby maximizing accuracy; (3) that PBMCs were stimulated/infected with the most common respiratory viruses and with ligands of TLRs that are representative of RNA respiratory viruses such as SARS-CoV-2; and (4) that stimulations were carried out using a single batch of each virus/stimulus and conducted by a single highly experienced individual, so any variability in response is likely participant-related and not a result of technical variability. The prevalence of hospital admissions due to LRTIs and RSV bronchiolitis in our cohort we report here is similar to that reported in England^27^, offering further reassurance about the generalizability. It is of note that the burden of respiratory diseases, including LRTI hospitalizations in infancy, is higher in Manchester compared to the rest of England. This is exemplified by the data on hospital admissions for bronchiolitis which has shown that Manchester has the highest hospital admission rates in England (approximately double that of the all-England data), with between 4% and 6.5% of infants in Manchester being hospitalized each year^27^, which is comparable with data in our cohort (6.44%).

The sex bias in enhanced production of IFN-α by TLR-7/8 stimulated PBMCs in females, which is attributed to an enhanced capacity of plasmacytoid dendritic cells (pDCs) to produce IFN-α in adults^28–32^ and pre- and post-pubertal subjects^33^ is well known. To our knowledge sex differences in IFN responses have not been studied previously with respiratory viruses as stimuli in a large population-based birth cohort in which relationships to severe clinical illnesses were studied. In our study TLR9 driven IFN-α production was also higher in adolescent females, which is a novel finding.

We acknowledge several limitations. We were not able to assess the importance of induction of the type III IFNs, IFN-λs 1–3, which are important in innate responses against respiratory viruses^34,35^, as the type III IFN, IFN-λ1 was not significantly induced by any infection/stimulus in the PBMCs that we studied. This is likely because respiratory epithelial cells are the main source of type III IFNs in the lung and because pDCs, which would be the main source of type III IFNs in freshly harvested PBMCs, which do induce type III IFNs in response to RV -A16 stimulation, were likely lost through the freeze/thaw process^36^.

We only studied IFN responses in samples taken at age 16. Further studies of IFN responses across the ages from birth/infancy through to old age, will be needed to more directly relate observed deficiencies with disease outcomes in different age groups.

The response of pDCs in PBMCs processed later than 6 h after collection is much attenuated compared to PBMCs isolated from freshly drawn blood^37^. We probably underestimated the production of IFN-α because the PBMCs we studied were frozen. However, this characteristic will be consistent in both groups, and it will not affect the comparisons between sexes.

There are alternative mechanisms that could explain sex difference in respiratory illnesses in younger children. For example a sex difference has been reported in numbers of cord blood type 2 innate lymphoid cells (ILC-2) with higher numbers in males compared to females^38^. The increased ILC-2 proportions in male neonates could be associated with increased Th2 responses and susceptibility to Th1-dependent infections in boys than in girls during childhood.

PBMCs were not directly stimulated with SARS-CoV-2, which limits the confidence whether our findings can be extended to COVID-19. However, we did study ligands to the endosomal expressed TLRs 7/8/9 (R848 and CpG-A), which are clearly engaged by positive strand RNA viruses such as SARS-CoV-2, which requires endosomal processing as part of viral entry into cells^39^. Furthermore, consistency of within-individual responses across all viral stimuli suggests that the observed differences are generic across multiple respiratory viruses (therefore likely including SARS-CoV-2), rather that virus-specific.

IFN-α and IFN-β are type I IFNs that are critical mediators of innate anti-viral immune responses, inducing apoptosis of virus-infected cells and inducing over 300 IFN-stimulated genes, many of which have a variety of direct anti-viral activities^40^. Through these combined activities, type I IFNs can abort virus replication in virus-infected cells^41^. IFN-γ is the only type II IFN, and it also is very important in promoting innate immune responses, principally by activating natural killer (NK) cells which are important in innate immune defense against virus infections by rapid killing of virus-infected cells^42^. IFN-γ also primes other immune cells such as macrophages, to release anti-viral cytokines^43^ and to phagocytose infected cells^42^. IFN-γ has been shown to suppress mouse coronavirus replication, though this was dependent, in part, on induction of type I IFN secretion^44^. Type I and II IFNs also work together to activate macrophages, NK cells, dendritic cells and T cells by enhancing cell activation, antigen presentation, cell trafficking, cell differentiation and proliferation, resulting in markedly enhanced innate and acquired antiviral immune effector function^45^. Thus, deficiency in either or both of these IFN types would be expected to increase the severity of virus-induced illnesses. We have previously described deficiency in IFN-α^46^, IFN-β^41,46^ and IFN-γ^47,48^ in patients with asthma and have reported that these patients have increased susceptibility to respiratory virus infections^49^. In addition, recent reports indicate that deficiencies in IFN responses are linked to increased susceptibility to virus-induced exacerbations of chronic obstructive pulmonary disease^50,51^. These data therefore support the biological and clinical relevance of our findings.

Can our findings be extended to the COVID-19 pandemic? IFN-α and IFN-β have both been shown to inhibit replication of SARS-CoV-2^52,53^. Importance of type I IFN immunity in COVID-19 has been highlighted by a study which demonstrated that neutralising autoantibodies against type I IFN (which were mostly found in older males) may be responsible for life-threatening COVID-19 pneumonia^54^, and evidence that deficiency in type I IFNs may be the key contributor to the development of severe disease, however this work did not consider the role of sex differences in COVID-19 severity^55^. Our data on the lower type I IFN responses to viral stimuli which mimic SARS-CoV-2 infection in males at age 16, coupled with recently reported association between life-threatening COVID-19 pneumonia and loss-of-function genetic variants at candidate loci involved in the induction and amplification of type I IFNs (which suggested a key role for type I IFN cell-intrinsic immunity in the control of SARS-CoV-2 infection)^56^ are consistent with epidemiolocal observations and, if replicated in studies in older persons, would provide a cogent explanation for the observed sex differences in COVID-19 severity. Furthermore, consistency of within-individual responses across all viral stimuli (Figure S3) and consistency of patterns of responses across all viral stimuli (Table 3) suggests that the observed differences are generic across multiple respiratory viruses (therefore likely including SARS-CoV-2), rather than virus-specific.

This interpretation is supported by a study which investigated sex differences in immune responses in patients hospitalised with COVID-19 and reported that female patients mounted significantly more robust T cell activation than males^57^, and a poor T cell response was associated with worse outcome in male patients. Robust innate IFN responses drive robust T cell responses during viral infections^45^, and deficient innate IFN responses studied ex vivo pre-infection are associated with increased virus load, higher inflammatory responses and worse clinical outcomes upon subsequent in vivo respiratory virus infection^34^. We therefore suggest that the above-described differences between sexes in T-cell activation in COVID-19^57^ may be a consequence of the deficient innate IFN responses in males that we report herein at age 16, being replicated in older subjects. Further studies in older persons will be needed to determine whether the sex bias in mortality and morbidity in older people with COVID-19 is indeed a consequence of deficient innate IFN responses in older males compared to older females.

Our data give pointers to the mechanistic framework to explain the observed differences between sexes. TLRs 7 and 8, which are engaged by positive strand RNA viruses and whose activation results in production of type I IFNs, are encoded by loci on the X chromosome. Bi-allelic expression of X-linked genes could enhance TLR7-8 expression in female immune cells^58^, thereby leading to the higher production of type I IFNs in females.

A recent study of self-reported cold frequency in adults with a median age in the early 30 s found no difference between sexes in cold frequency, or in whole blood TLR7 and TLR8 gene expression^59^. Interestingly, female participants had higher IFN-α induction in PBMCs 24-h post stimulation with RV-A16 than males, which is in line with our findings.

Our findings have implications for the prevention and therapy of respiratory virus infections, which are best implemented when high-risk populations can be identified. Respiratory viruses are initially encountered by the nasal epithelium where infection is initiated. An adequate anti-viral response can contain the virus in the upper airway, with cold-like symptoms only, and a diminished response can see the infection spreading to the lower airways to induce severe LRTI. Our data support a preventive strategy, particularly among males, which involves targeting the innate immune system with ‘immune training’ agents to boost resistance to primary infection and enhance the capacity to control the intensity of airways’ inflammatory responses^60^. Our data also suggest that therapeutic use IFNs, or of agents that boost innate IFN induction by virus infections, may be efficacious in treatment. This is supported by evidence from randomised controlled trials which have reported benefits of inhaled nebulised IFN-β as a treatment of virus-induced worsening of asthma in difficult-to-treat asthma^61^, and of patients admitted to hospital with severe COVID-19^62^. Consistent with this is a recent report indicated that early subcutaneous IFN-β administered up to three times (with ribavirin) in the first seven days after diagnosis of COVID-19 was associated with faster virus clearance and substantial clinical improvement with shorter hospital stays^63^. It is likely that most of this clinical benefit resulted from IFN-β rather than ribavirin^64^. A further clinical trial demonstrated that early administration of IFN-β1a in severe COVID-19 patients significantly increased discharge rates on day 14 and decreased 28-day mortality^65^.

Our findings have important implications for another hotly debated topic, namely whether “man flu” actually exists or not^66,67^. Here we provide a biological basis to explain why males would be expected to experience more severe disease than females when infected by respiratory viruses.

In conclusion, high morbidity and mortality from respiratory viruses among males is likely explained by impaired innate anti-viral immune responses in males compared to females. The marked reductions in type I IFN responses to viral stimuli reported herein in males, coupled with the relationships with virus-induced exacerbations of lung disease, and with severe viral LRTIs among male participants and among those with the most deficient IFN induction in our cohort, indicate that the deficient IFN responses we report in males may be in part responsible for the adverse outcomes to SARS-CoV-2 infection currently being reported in males. These findings have important clinical implications and call urgently for further studies of IFNs and IFN-inducing therapies, studied when given early in the disease course, when benefit is most likely to be significant^62,63^, as IFN administration late in the disease course in the Solidarity trial demonstrated no benefit and tended towards harm^68^.

## Methods

### Study design, setting, participants and data sources

The study subjects were participants in the Manchester Asthma and Allergy Study (MAAS), a population-based birth cohort study^69^. The study was approved by a Manchester Local Research Ethics Committee (ERP/94/032, approval received on 24/03/1994; SOU/00/258 and SOU/00/259, approval received on 20/12/2000; 03/SM/400, approval received on 05/12/2003; Study registration ISRCTN72673620, http://www.isrctn.com/ISRCTN72673620). Participants were recruited from the maternity catchment area of Wythenshawe and Stepping Hill Hospitals (50 square miles of South Manchester and Cheshire), a stable mixed urban–rural population (http://www.maas.org.uk). Written informed consent was obtained from parents. All methods were performed in accordance with the relevant guidelines and regulations.

All pregnant women were screened for eligibility at antenatal visits (8th-10th week of pregnancy) between 1995 and 1997. Of the 1499 couples who met the inclusion criteria (< 10 weeks of pregnancy, maternal age > 18 years), 288 declined to take part and 27 were lost to follow-up between recruitment and birth of a child. A total of 1184 participants had some evaluable data. We used male or female sex as assigned at birth. Children have been followed prospectively, and attended review clinics at ages 1, 3, 5, 8, 11 and 16 years. We carried out home visits for study participants who could not attend clinic appointments.

We extracted data from General Practitioner (GP)-held medical records including prescriptions, acute episodes, medication prescriptions and hospital admissions. A trained physician reviewed the written and computerized primary care medical records for each child. All consultations with health care providers including hospital admissions, hospital outpatient visits and use of the out of hours services, with linked prescriptions (drug name, route of administration and the dose) was separately entered by the date of the event, allowing the calculation of child’s age in days at each event. Information captured included location, the type of visit, reason for that particular consultation and any relevant symptoms and diagnoses (such as LRTI or bronchiolitis).

### Definition of clinical outcomes

*Younger and older siblings*: Presence or absence of younger and older siblings.

*Maternal and paternal asthma*: If parents of the individual ever suffered from asthma.

*Current wheeze*: Positive answer to the question “Has your child had wheezing or whistling in the chest in the last 12 months?”.

*Current asthma at age 16 years*: Defined as the presence of any two of the following three features: (1) Current wheeze; (2) Current use of asthma medication; and (3) Physician-diagnosed asthma ever.

*Lower respiratory tract infections*: We extracted data on severe LRTIs requiring hospital admissions in early life from primary care medical records; age in days at the time of each event was documented to provide an accurate account of each episode^24^.

### Cell stimulations and cytokine measurement

We collected, processed and cryopreserved peripheral blood mononuclear cells (PBMCs) at age 16 years in all children who agreed to provide blood samples.

*PBMC isolation*: ~ 8 mL of venous blood was collected into a heparinised tube. PBMCs were separated by centrifugation over Ficoll-Hypaque. Cell viability was determined using trypan blue and cells were resuspended in freezing medium (15% DMSO in Heat Inactivated Foetal Calf Serum) at 10^6^ cells/mL and cryopreserved in liquid nitrogen.

#### Cell stimulation

Cryobanked PBMCs were shipped for cell stimulations. On the day of experiment PBMCs were thawed on the day of stimulation, counted and had viability checked as previously described^23,24^. We used RPMI1640 with L-glutamine, HEPES, Na bicarbonate, penicillin/streptomycin and 10% foetal bovine serum as medium control. Cells were distributed in 96-well round bottom plates at 2*10^5^ cells/well and were stimulated with two live rhinoviruses (RVs), RV-A16 and RV-A1, and live respiratory syncytial virus (RSV) all at a multiplicity of infection (MOI) of 1. After an hour of incubation with the live viruses, the viral inoculum was removed and 200 μL of fresh control medium added. The cells were returned to the incubator and harvested 24 h post-stimulation/infection. All viruses were originally obtained from the American Type Culture Collection (ATCC). We also used two viral mimics (which mimic infection with SARS-CoV-2), the Toll-like receptor (TLR)-7/8 ligand resiquimod (R848) and the TLR-9 ligand class A CpG oligonucleotide (CpG-A), both at 1 μM concentrations (Invivogen).

#### Cytokine measured

Protein concentrations of 27 cytokines were measured in supernatants 24 h post-stimulation, using the Meso Scale Discovery® multiplex kits (http://www.mesoscale.com) as previously described^23,24^. For the present analyses we focussed on the three IFNs: IFN-α, IFN-β, IFN-γ and four IFN-induced chemokines CXCL10/IP-10, CCL2/MCP1, CCL4/MIP-1β and CCL13/MCP4 that were significantly induced by each of the viruses/stimuli studied. Samples below the lower detection limit of the assay were assigned a value of ½ the lower detection limit.

### Data pre-processing

#### Quality control

Prior to analyses, data were pre-processed according to the pipeline described in our previous study^23^, to exclude samples with low viability and/or no response of any cytokine to any stimulus. We excluded 13 samples with cell viability < 5% upon thawing. Samples meeting all the following criteria were also excluded as inadequate responders: viability < 20% and interleukin (IL)-2 response to phytohemagglutinin < 5 pg/mL and interferon (IFN)-α response to respiratory syncytial virus (RSV) < 5 pg/mL and IFN-γ response to rhinovirus (RV)-A16 < 7 pg/mL and IL-6 response to *Hemophilus influenzae* < 5 pg/mL (n = 2). These methods were exactly as used in our previous studies on this cohort using PBMCs collected at age 11 years^23,24^. One child was excluded with 24% viability, as no cytokine response was seen to any stimulus. After quality control excluded these 16 children, we had data from 345 participants for the analysis. IFN-γ responses for some subjects were lost due to an MSD plate malfunction (Fig. 1). To achieve data normalisation, we subtracted the log-transformed media response for each cytokine from the log-transformed cytokine responses to stimulation. We used these transformed values for the evaluation of cytokine responses.

### Statistical analysis

Differences in clinical outcomes (hospital admissions with LRTIs and bronchiolitis) were assessed through χ^2^ and Fishers exact test, and logistic regression analysis using males as a reference group.

We expressed the induction of IFNs and IFN-induced chemokines as raw values (in pg/mL) and fold-induction over medium controls. The values were computed as the log(x) − log(media) = log(x/media). The data were summarized as median and interquartile range (IQR). Univariate comparisons between groups were performed using the Wilcoxon rank sum test (2-tailed). Benjamini–Hochberg correction was applied to account for multiple testing^70^. Proportions of males and females with IFN-α responses to viral stimuli below the 15th, 20th and 25th percentiles of the entire population and frequencies of hospital admissions with LRTIs and bronchiolitis in particpants above and below the 15th percentile of the entire population of IFN-α responses to viruses were compared using χ^2^ tests. Associations with *P* < 0.05 were considered significant.

## Supplementary Information

Supplementary Information.

## Data Availability

The data that support the findings of this study are available from the public depository, Zenodo, https://zenodo.org/record/4811813#.YK5I85NKj0o).
