# Supplementary material for: Sex differences in innate anti-viral immune responses to respiratory viruses and in their clinical outcomes in a birth cohort study
Source: Sci Rep. 2021 Dec 9;11:23741. doi: 10.1038/s41598-021-03044-x (PMC8660814; doi:10.1038/s41598-021-03044-x)
Supplement: Supplementary file 1 — Supplementary Information. [file 41598_2021_3044_MOESM1_ESM.pdf]

Sex differences in innate anti-viral immune responses to respiratory viruses and in their clinical outcomes in a birth cohort study

Eteri Regis[1#], Sara Fontanella[1#], Lijing Lin[2], Rebecca Howard[2], Sadia Haider[1], John A. Curtin[3], Michael R. Edwards[1], Magnus Rattray[2], Angela Simpson[3], Adnan Custovic[1†], Sebastian L. Johnston[1*†]

[1]National Heart and Lung Institute, Imperial College London, United Kingdom

[2]Faculty of Biology, Medicine and Health, University of Manchester, M13 9PT, UK

[3]Division of Infection, Immunity and Respiratory Medicine, Faculty of Biology, Medicine and Health, Manchester Academic Health Sciences Centre, University of Manchester and University Hospital of South Manchester NHS Foundation Trust, Manchester, UK

Author Contributions:

[#]Eteri Regis and Sara Fontanella contributed equally to this article

[†]Adnan Custovic and Sebastian Johnston contributed equally to this article

Supplementary Appendix

This PDF file includes:

Figures S1 to S4

Tables S1 to S3

SUPPLEMENTARY FIGURES AND TABLES

Figure S1. Cell viability between sexes.

Data were analysed using the Wilcoxon test. Each dot represents an individual participant. Box plots represent the 25th and 75th percentiles, the line the median, with whiskers at the 10th and 90th percentiles. Data are presented in percentages.

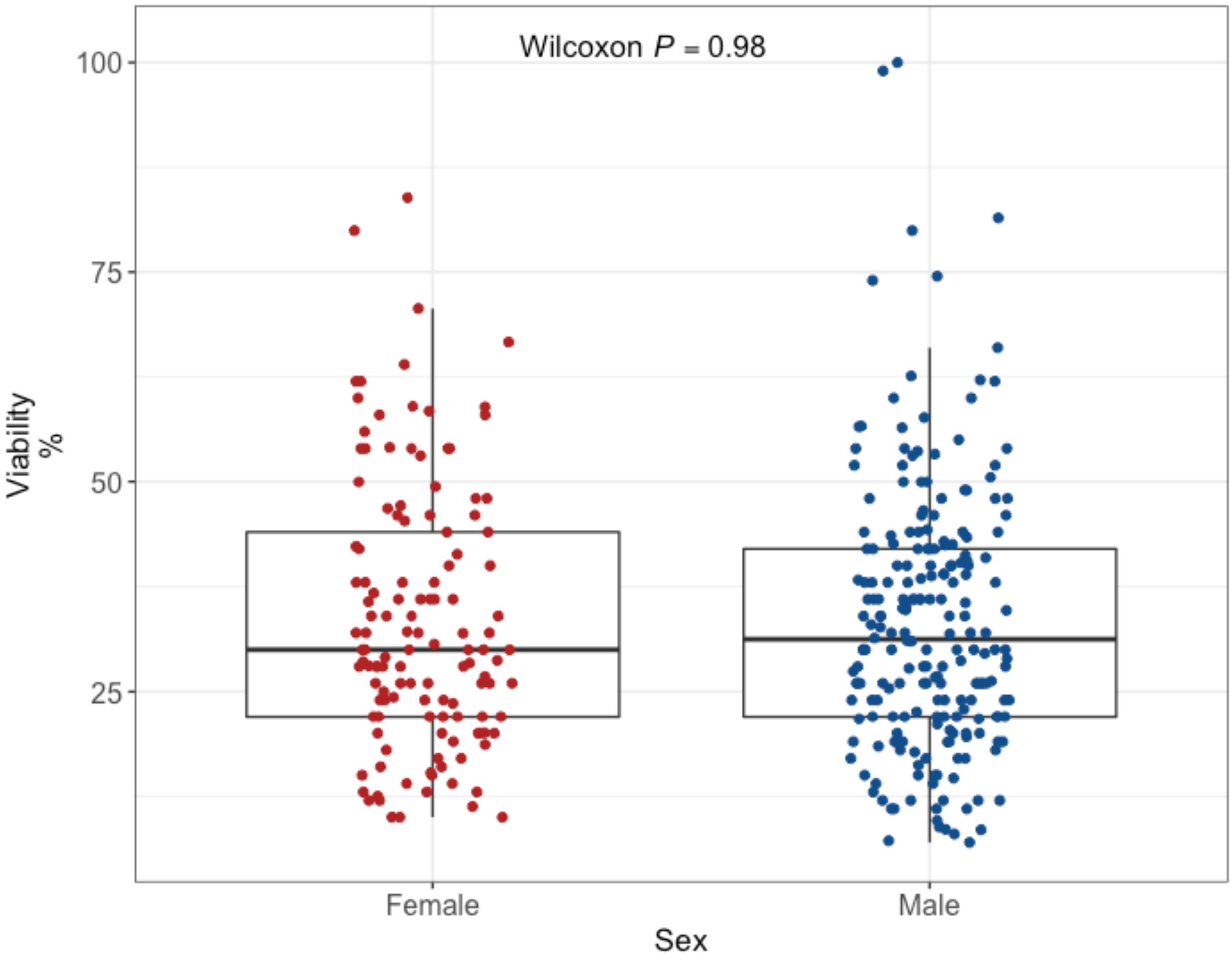

Figure S2. Sex differences in IFN-α, CCL4/MIP-1β, CXCL10/IP-10 and CCL13/MCP4 induction in response to RV-A1 and RSV induction.

Box plots represent the 25th and 75th percentiles, the line the median, with whiskers at the 10th and 90th percentiles. Each dot represents an individual participant. Wilcoxon test: a) *P*=0.011, b) *P*=0.041, C) *P*=0.010, d) *P*=0.002, e) *P*=0.018 and f) *P*=0.019. Data are presented as fold induction. The y axis is plotted on a logarithmic scale.

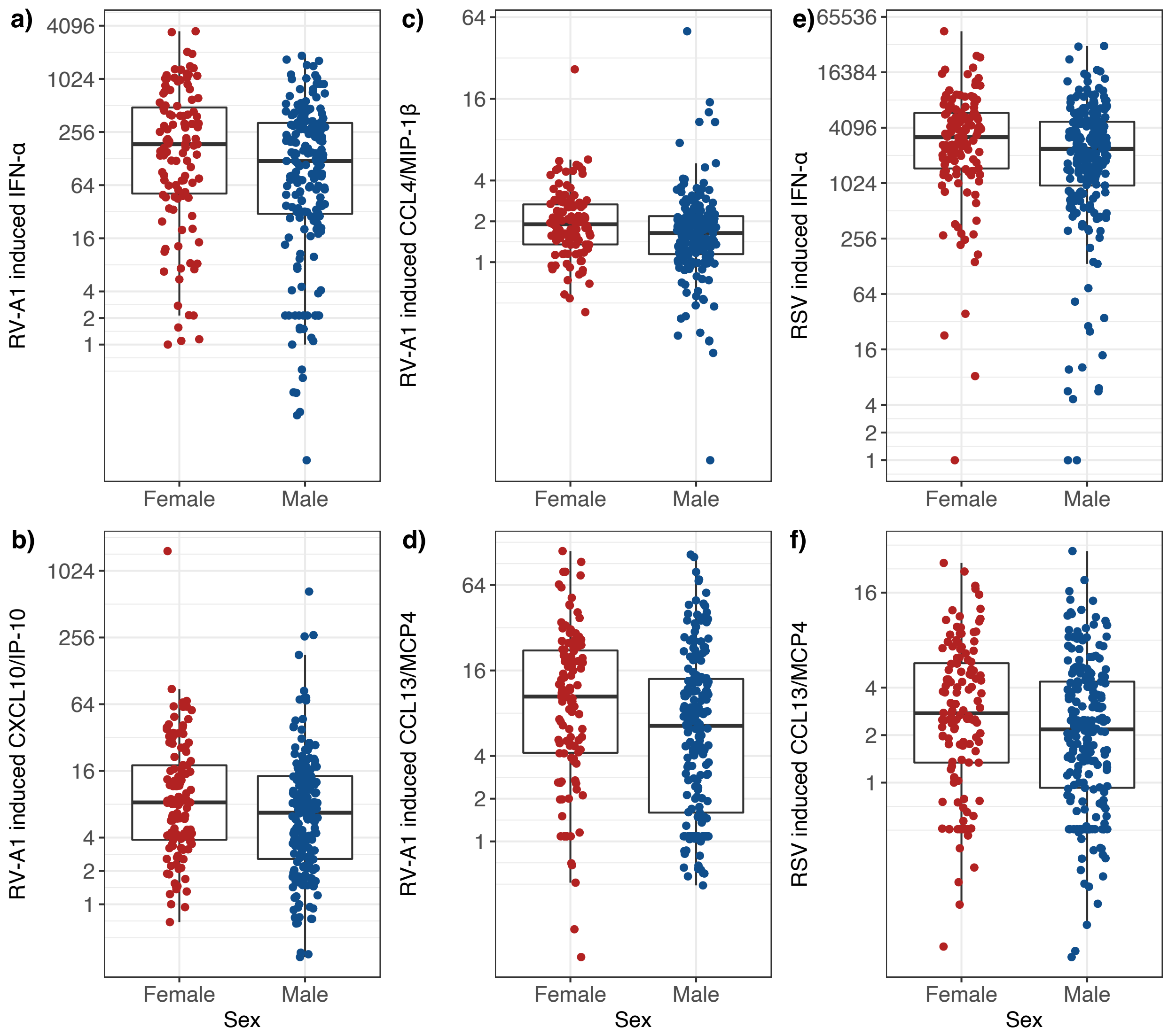

Figure S3. Individual IFN-α responses of PBMCs after stimulation with three respiratory viruses and two viral mimics

Alluvial plots of the individual IFN-α responses across the five stimuli (for participants with IFN-α data for all five stimuli). LL: 1st quartile; ML: 2nd quartile; MH: 3rd quartile; HH: 4th quartile determined on the whole population.

A)

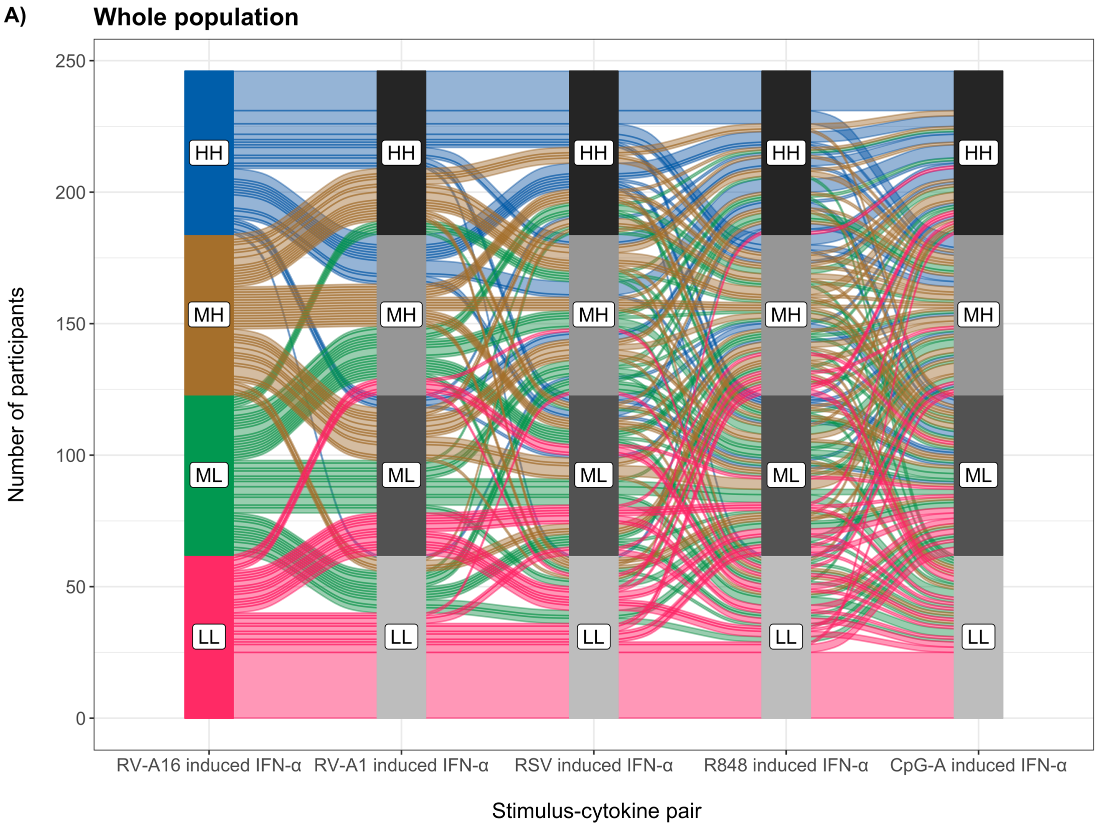

B)

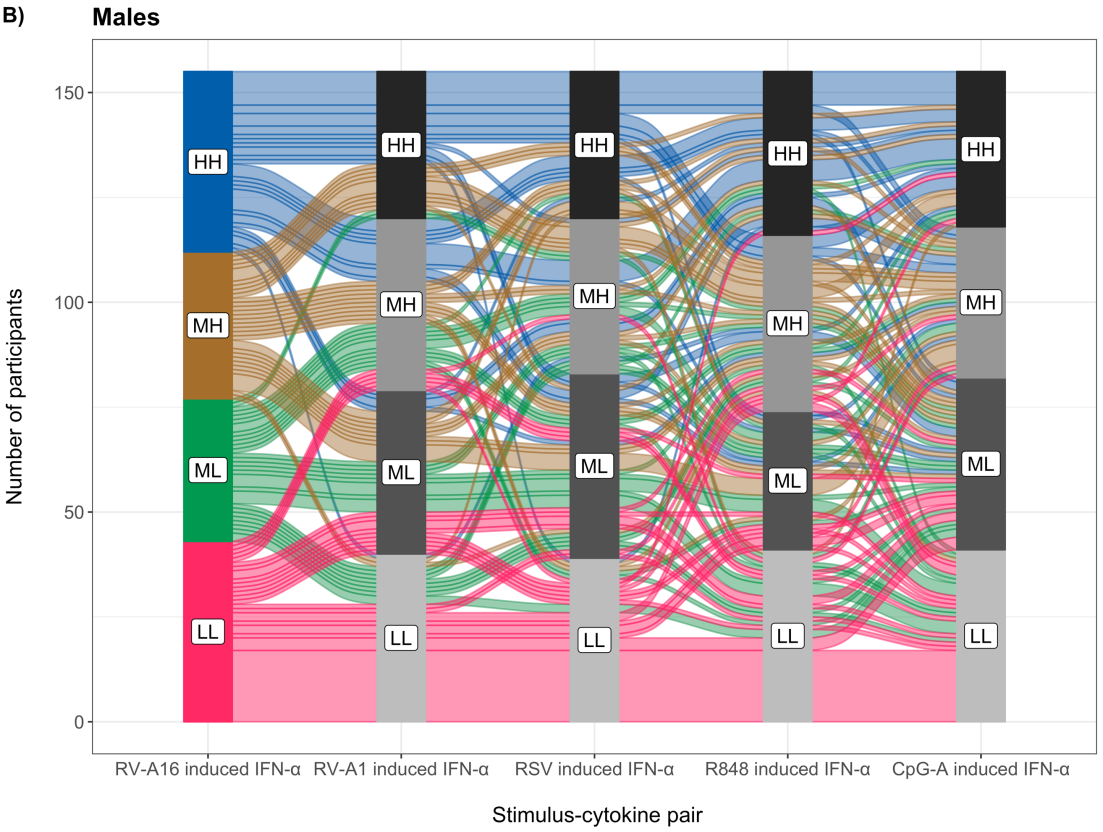
Males
Number of participants
150
100
50
0
HH
MH
ML
LL
RV-A16 induced IFN-α
RV-A1 induced IFN-α
RSV induced IFN-α
R848 induced IFN-α
CpG-A induced IFN-α
Stimulus-cytokine pair

C)

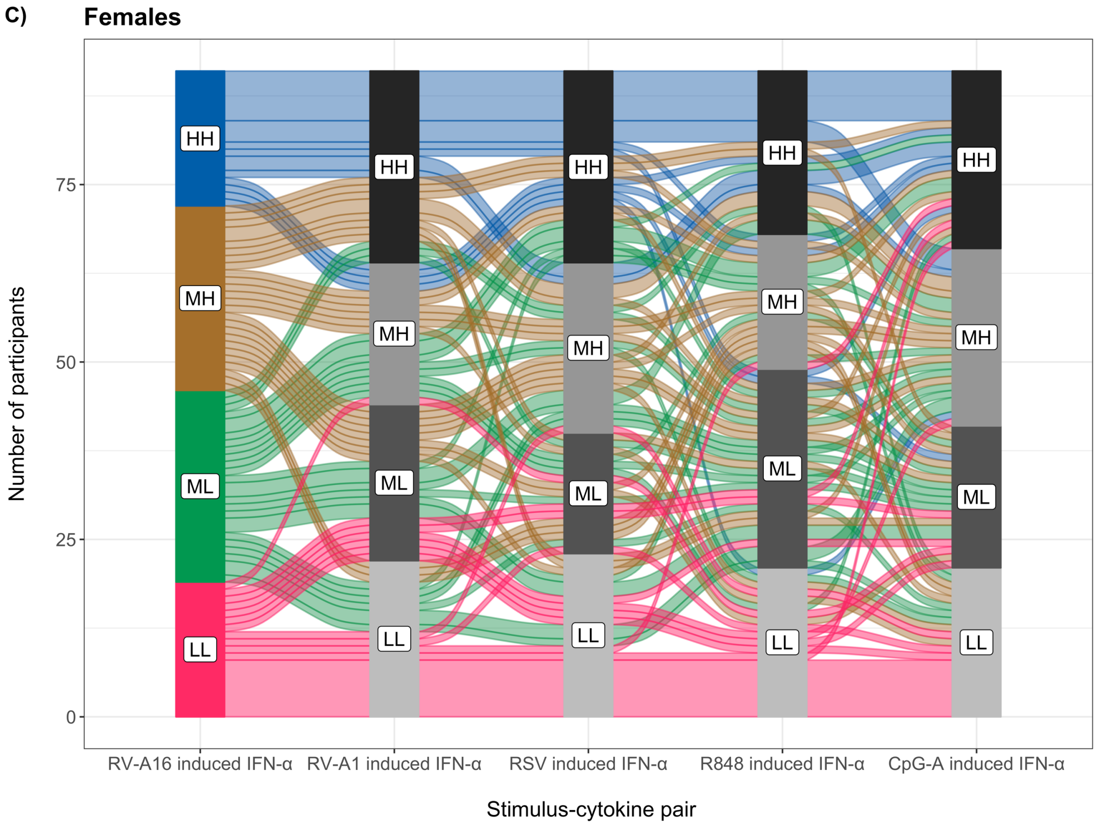
Females
Number of participants
75
50
25
0
HH
MH
ML
LL
RV-A16 induced IFN-α
RV-A1 induced IFN-α
RSV induced IFN-α
R848 induced IFN-α
CpG-A induced IFN-α
Stimulus-cytokine pair

Figure S4. Proportions of males and females with IFN-α responses to CpG-A and RV-A1 below the 15th, 20th and 25th percentiles of the entire population.

P-values are derived using chi-squared tests.

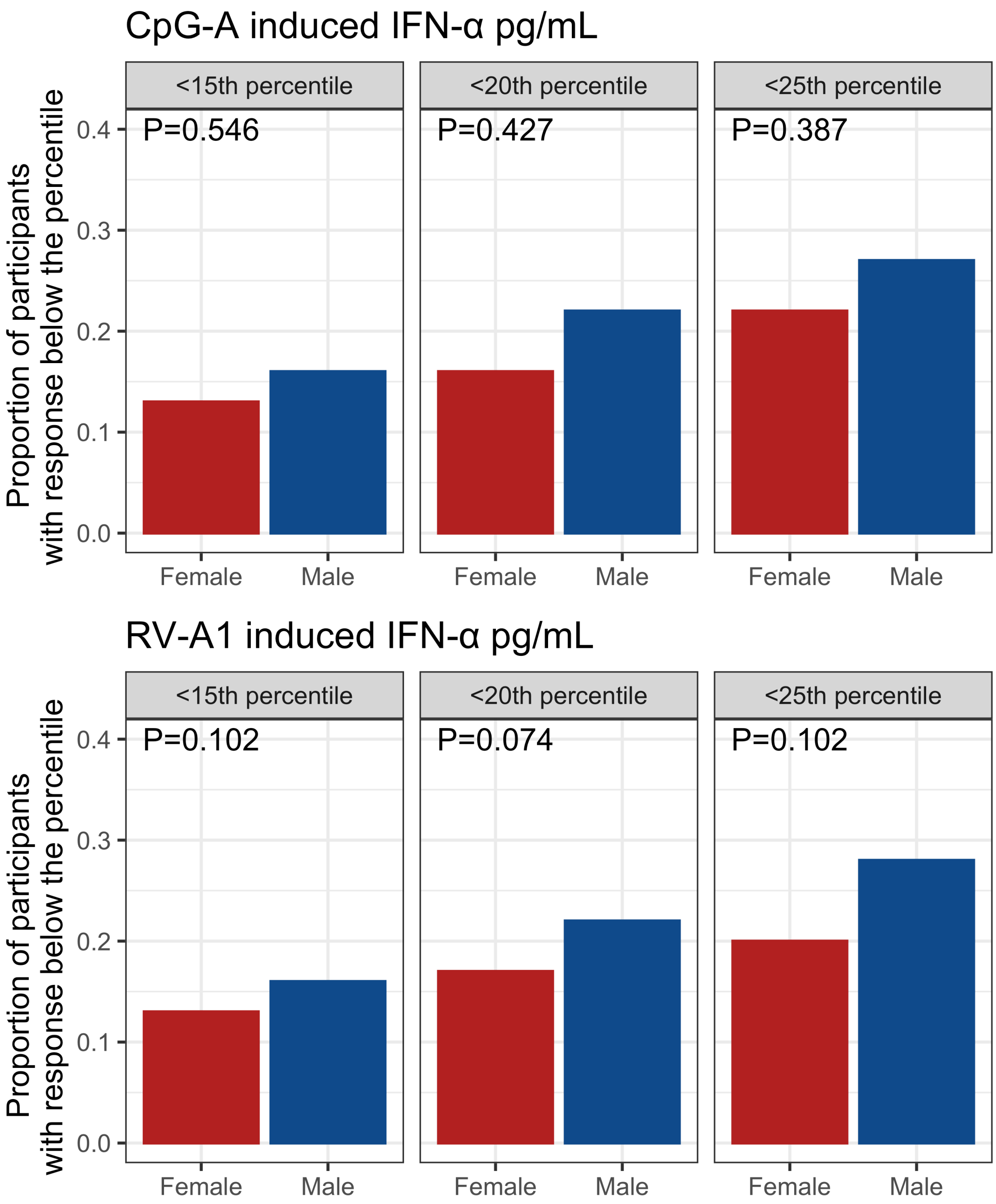

Table S1. Comparisons of lower respiratory tract infection (LRTI) hospital admissions, bronchiolitis and RSV positive bronchiolitis cases in female and male participants with cytokine data.

Differences were assessed through χ^2 and *Fishers exact test* for sample size >5 and ≤ 5, respectively.

	**Female (n=116)**	**Male (n=195)**	***P*-value**
LRTI admission in the first year of life	8 (6.9%)	18 (9.2%)	0.472
LRTI admission in the second year of life	1 (0.9%)	10 (5.1%)	0.059
LRTI admission in the first two years of life	9 (7.8%)	26 (13.3%)	0.142
LRTI admission by third year of life	12 (10.3%)	31 (15.9%)	0.180
Ever hospitalised for LRTI age 0-8	15 (12.9%)	35 (17.9%)	0.244
RSV positive bronchiolitis	2 (1.7%)	8 (4.1%)	0.331
Ever bronchiolitis	3 (2.6%)	16 (8.2%)	0.045

Table S2. Comparisons of demographic and clinical characteristics between children with and without PBMC stimulation data at age 16 years stratified by sex. Differences were assessed through χ^2 and *t*-test for categorical and continuous variables, respectively.

	Female (n=353)			Male (n=398)		
	**Cytokine data (n=128)**	**No Cytokine data (n=225)**	***P*-value**	**Cytokine data (n=217)**	**No Cytokine data (n=181)**	***P*-value**
	**n (%)**	**n (%)**	χ^2	**n (%)**	**n (%)**	χ^2
Ethnicity - Caucasian	121/126 (96)	212/220 (96.4)	0.925	204/212 (96.2)	165/171 (96.5)	0.657
Younger siblings	70/128 (54.7)	113/225 (50.2)	0.420	112/217 (51.6)	89/181 (49.2)	0.628
Older siblings	62/128 (48.4)	108/221 (48.9)	0.938	121/215 (56.3)	94/180 (52.2)	0.420
Day care attendance	91/121 (75.2)	144/219 (65.8)	0.710	153/209 (73.2)	115/163 (70.6)	0.572
Maternal smoking (pregnancy)	9/128 (7.0)	28/217 (12.9)	0.089	19/212 (9)	15/178 (8.4)	0.851
Maternal smoking (current)	10/126 (7.9)	27/225 (12.0)	0.234	29/217 (13.4)	20/181 (11.0)	0.484
Maternal asthma	23/128 (18.0)	53/225 (23.6)	0.220	39/217 (18.0)	33/181 (18.2)	0.947
Paternal asthma	22/128 (17.2)	40/225 (17.8)	0.889	29/217(13.4)	18/181 (9.9)	0.293
Dog ownership	40/127 (31.5)	84/225 (37.3)	0.271	80/216 (37.0)	71/180 (39.4)	0.623
Cat ownership	29/127 (0.3)	64/225 (28.4)	0.252	54/215 (24.1)	40/177 (22.6)	0.561
Current asthma	20/126 (15.9)	34/223 (15.2)	0.876	39/213 (18.3)	40/179 (22.3)	0.321
Current wheeze	18/126 (14.3)	38/225 (16.9)	0.523	33/215 (15.3)	38/180 (21.1)	0.137
Current rhinitis	43/127 (33.9)	86/224 (38.4)	0.397	96/217 (44.2)	84/181 (46.4)	0.665
LRTI admissions	9/116 (6.7)	13/193 (7.8)	0.820	26/195 (13.3)	29/147 (19.7)	0.137
RSV-positive bronchiolitis	2/116 (1.7)	3/193 (1.6)	1.000	8/195 (4.1)	7/147 (4.8)	0.795
	**Mean (SD)**	**Mean (SD)**	***t*-test**	**Mean (SD)**	**Mean (SD)**	***t*-test**
Age at follow up	16.0 (0.66)	16.1 (0.50)	0.698	16.1 (0.55)	15.9 (0.78)	0.085
Birth weight (kg)	3.42 (0.47)	3.32 (0.91)	0.213	3.51 (0.93)	3.57 (0.54)	0.396

Table S3. Differences between males and females in IFN and IFN-induced chemokine concentrations.

Log2 transformed cytokine concentration data (in pg/mL) were analysed using the Wilcoxon test. *P* values and adjusted *P* values less than 0.05 are in bold. The group with the higher IFN and IFN-induced chemokine concentration is highlighted in grey.

Stimulus	Cytokine	Median concentration [IQR]	Median concentration [IQR]	*P* value	Adjusted *P* value	Female to Male ratio in fold induction
RV-A16		**Female, n=128 (37.2%)**	**Male, n=216 (62.8%)**			
	IFN-α	6.65 [5.82 - 7.74]	5.94 [4.63 - 6.98]	**0.000**	**0.001**	0.710
	IFN-β	4.63 [3.31 - 5.87]	3.71 [0.51 - 5.17]	**0.001**	**0.005**	0.920
	IFN-γ	7.72 [5.9 - 8.64]	6.54 [5.07 - 8.01]	**0.000**	**0.005**	1.180
	CCL4/MIP-1β	12.07 [11.6 - 12.63]	12.11 [11.48 - 12.66]	0.815	0.878	-0.040
	CXCL10/IP-10	11.75 [10.78 - 12.39]	11.11 [10.12 - 12.02]	**0.002**	**0.018**	0.640
	CCL13/MCP4	7.4 [5.7 - 8.19]	6.89 [5.77 - 8.19]	0.276	0.475	0.510
	CCL2/MCP1	13.46 [12.4 - 13.94]	13.16 [12.09 - 13.91]	0.132	0.325	0.300
RSV		**Female n=128 (37.1%)**	**Male n=217 (62.9%)**			
	IFN-α	8.11 [7.19 - 8.83]	7.81 [6.84 - 8.71]	0.089	0.267	0.300
	IFN-β	5.41 [4.48 - 5.99]	5.22 [4.37 - 6.16]	0.621	0.776	0.190
	IFN-γ	6.88 [5.63 - 8.06]	6.51 [4.66 - 7.8]	**0.031**	0.147	0.370
	CCL4/MIP-1β	11.47 [10.71 - 12.5]	11.57 [10.64 - 12.49]	0.953	0.958	-0.100

	CXCL10/IP-10	11.05 [10.29 - 11.74]	10.86 [10.11 - 11.63]	0.183	0.388	0.190
	CCL13/MCP4	6.15 [5.18 - 6.92]	5.96 [4.93 - 6.7]	0.139	0.325	0.190
	CCL2/MCP1	12.9 [11.99 - 13.66]	12.67 [11.8 - 13.46]	0.207	0.388	0.230
RV-A1		**Female n=115 (37.2%)**	**Male n=194 (62.8%)**			
	IFN-α	3.97 [2.42 - 5.33]	3.82 [1.72 - 4.8]	0.076	0.247	0.150
	IFN-β	-0.27 [-0.27 - 2.07]	-0.27 [-0.27 - 1.69]	0.508	0.727	0.000
	IFN-γ	5.76 [4.05 - 7.57]	5.02 [3.67 - 6.5]	**0.028**	0.147	0.740
	CCL4/MIP-1β	10.76 [9.75 - 11.98]	10.72 [9.52 - 11.83]	0.757	0.874	0.040
	CXCL10/IP-10	10.46 [9.38 - 11.4]	10.16 [9.23 - 11.24]	0.096	0.268	0.300
	CCL13/MCP4	8.11 [6.74 - 8.75]	7.45 [5.96 - 8.62]	**0.032**	0.147	0.660
	CCL2/MCP1	13.2 [12.21 - 13.92]	12.96 [11.83 - 13.8]	0.203	0.388	0.240
R848		**Female n=102 (37.6%)**	**Male n=169 (62.4%)**			
	IFN-α	6.4 [5.13 - 7.76]	5.58 [4.25 - 6.77]	**0.000**	**0.005**	0.820
	IFN-β	0.04 [0.04 - 2.26]	0.04 [0.04 - 1.98]	0.127	0.325	0.000
	IFN-γ	7.64 [6.03 - 8.99]	6.99 [5.54 - 8.54]	0.063	0.234	0.650
	CCL4/MIP-1β	13.55 [12.82 - 14.26]	13.54 [12.92 - 14.3]	0.536	0.727	0.010
	CXCL10/IP-10	9.7 [9.2 - 10.28]	9.5 [9.08 - 10.08]	0.065	0.234	0.200
	CCL13/MCP4	5.55 [4.6 - 6.22]	5.41 [4.18 - 6.39]	0.773	0.874	0.140

	CCL2/MCP1	13.51 [12.61 - 14.24]	13.42 [12.63 - 14.09]	0.628	0.776	0.090
CpG-A		**Female n=91 (36.9%)**	**Male n=156 (63.1%)**			
	IFN-α	8.88 [7.94 - 10.13]	8.6 [7.17 - 10.04]	0.209	0.388	0.280
	IFN-β	5.02 [2.46 - 6.55]	4.63 [-0.08 - 6.12]	0.212	0.388	0.390
	IFN-γ	3.87 [1.87 - 5.17]	2.76 [0.46 - 4.14]	**0.005**	**0.031**	1.110
	CCL4/MIP-1β	11.18 [10.25 - 12.3]	11.44 [10.32 - 12.31]	0.336	0.542	-0.260
	CXCL10/IP-10	11.42 [10.57 - 12.19]	11.23 [10.28 - 12.12]	0.482	0.724	0.190
	CCL13/MCP4	7.11 [6.09 - 7.98]	7.08 [6.01 - 7.81]	0.615	0.776	0.030
	CCL2/MCP1	12.68 [11.92 - 13.68]	12.81 [11.88 - 13.61]	0.958	0.958	-0.130
Media		**Female, n=128 (37.1%)**	**Male, n=217 (62.9%)**			
	IFN-α	-3.76 [-3.76 - -3.76]	-3.76 [-3.76 - -3.24]	0.067	0.234	0.000
	IFN-β	-1.49 [-1.49 - -1.49]	-1.49 [-1.49 - -1.49]	0.867	0.910	0.000
	IFN-γ	-1.08 [-1.08 - 0.29]	-1.08 [-1.08 - 0.1]	0.400	0.621	0.000
	CCL4/MIP-1β	9.63 [8.52 - 10.87]	9.74 [8.59 - 11.05]	0.521	0.727	-0.110
	CXCL10/IP-10	7.43 [6.2 - 8.72]	7.68 [6.38 - 8.58]	0.701	0.841	-0.250
	CCL13/MCP4	3.87 [3.87 - 5.18]	4.19 [3.87 - 5.4]	0.283	0.475	-0.320
	CCL2/MCP1	10.85 [9.82 - 12.2]	11.05 [9.57 - 12.11]	0.791	0.874	-0.200
